# Rewarming With Closed Thoracic Lavage Following 3-h CPR at 27°C Failed to Reestablish a Perfusing Rhythm

**DOI:** 10.3389/fphys.2021.741241

**Published:** 2021-09-29

**Authors:** Joar O. Nivfors, Rizwan Mohyuddin, Torstein Schanche, Jan Harald Nilsen, Sergei Valkov, Timofei V. Kondratiev, Gary C. Sieck, Torkjel Tveita

**Affiliations:** ^1^Anesthesia and Critical Care Research Group, Department of Clinical Medicine, UiT-The Arctic University of Norway, Tromsø, Norway; ^2^Department of Physiology and Biomedical Engineering, Mayo Clinic, Rochester, MN, United States; ^3^Division of Surgical Medicine and Intensive Care, University Hospital of North Norway, Tromsø, Norway; ^4^Department of Research and Education, Norwegian Air Ambulance Foundation, Drøbak, Norway

**Keywords:** accidental hypothermia, hypothermic cardiac arrest, organ blood flow, reperfusion, cardiopulmonary resuscitation

## Abstract

**Introduction:** Previously, we showed that the cardiopulmonary resuscitation (CPR) for hypothermic cardiac arrest (HCA) maintained cardiac output (CO) and mean arterial pressure (MAP) to the same reduced level during normothermia (38°C) vs. hypothermia (27°C). In addition, at 27°C, the CPR for 3-h provided global O_2_ delivery (DO_2_) to support aerobic metabolism. The present study investigated if rewarming with closed thoracic lavage induces a perfusing rhythm after 3-h continuous CPR at 27°C.

**Materials and Methods:** Eight male pigs were anesthetized, and immersion-cooled. At 27°C, HCA was electrically induced, CPR was started and continued for a 3-h period. Thereafter, the animals were rewarmed by combining closed thoracic lavage and continued CPR. Organ blood flow was measured using microspheres.

**Results:** After cooling with spontaneous circulation to 27°C, MAP and CO were initially reduced by 37 and 58% from baseline, respectively. By 15 min after the onset of CPR, MAP, and CO were further reduced by 58 and 77% from baseline, respectively, which remained unchanged throughout the rest of the 3-h period of CPR. During CPR at 27°C, DO_2_ and O_2_ extraction rate (VO_2_) fell to critically low levels, but the simultaneous small increase in lactate and a modest reduction in pH, indicated the presence of maintained aerobic metabolism. During rewarming with closed thoracic lavage, all animals displayed ventricular fibrillation, but only one animal could be electro-converted to restore a short-lived perfusing rhythm. Rewarming ended in circulatory collapse in all the animals at 38°C.

**Conclusion:** The CPR for 3-h at 27°C managed to sustain lower levels of CO and MAP sufficient to support global DO_2_. Rewarming accidental hypothermia patients following prolonged CPR for HCA with closed thoracic lavage is not an alternative to rewarming by extra-corporeal life support as these patients are often in need of massive cardio-pulmonary support during as well as after rewarming.

## Introduction

Accidental hypothermia is defined as an involuntary drop in core body temperature to <35°C. Mild (35–32°C) hypothermia is common involving no serious medical concerns (Danzl and Pozos, 1994; Walpoth et al., 2017). However, a severe (28–24°C) accidental hypothermia has been termed an “orphan disease,” since it is uncommon and can be life-threatening. In the United States, accidental hypothermia causes 1,500 deaths per year, approximating to 0.5 death per 100,000 inhabitants per year (Brown et al., 2012).

During exposure to low temperature, the thermogenesis and behavioral responses serve to minimize heat loss but these can be overwhelmed by the cold stress, and the core body temperature will continue to fall unless homeostasis is restored. During hypothermia, the coordinated thermoregulatory systems begin to fail, resulting in further heat loss (Danzl and Pozos, 1994; Zafren et al., 2014). The marked reduction in metabolism hypothermia is the main reason why successful resuscitation can be achieved after the prolonged periods in hypothermic cardiac arrest (HCA) (Weiss et al., 2012; Hilmo et al., 2014). As core body temperature decreases, HCA eventually occurs and is the main cause of the high mortality seen in severe and profound (<24°C) hypothermia (Lloyd, 1996). In mild hypothermia, thermoregulatory mechanisms are preserved. Thus, shivering is triggered to elevate the metabolic thermogenesis in the early phases of cooling and during mild hypothermia. However, at moderate hypothermia thermoregulatory mechanisms are lost (Giesbrecht, 2001). As core body temperature drops below 28°C, the risk of HCA increases substantially (Danzl and Pozos, 1994). The decreased sensitivity of central CO_2_-receptors leads to hypoventilation and respiratory acidosis (Zafren et al., 2014). However, the hypoxic respiratory drive is preserved until ~25°C when apnea eventually occurs (Young et al., 1995). At some point, irreversible hypothermia occurs (Lloyd, 1996), but survival and full neurological recovery have been reported in cases where core body temperature was as low as 11.8°C (Mroczek et al., 2020).

The current gold standard for rewarming hypothermic patients with unstable circulation or in HCA is by use of extra-corporeal life support (ECLS), applying any form of cardio-pulmonary bypass, and there is clinical consensus that these patients should be transported to a ECLS-facility for rewarming (Brown et al., 2012; Brugger et al., 2013; Zafren et al., 2014). The rationale for active internal (or central) rewarming is that it is more effective (Lloyd, 1996) and may reduce the risk of severe rewarming shock, i.e., decline in systemic vascular resistance not associated with an increase in cardiac output (CO) (Brunette et al., 1987). In 2014, a systematic review evaluated ECLS used to rewarm hypothermia in HCA and revealed a 67.7% survival to discharge and a 61.5% rate of good neurological recovery (Dunne et al., 2014). However, there are limited data available for comparison of patients with profound or severe hypothermia in cardiac arrest that were not treated with ECLS (Brown et al., 2012).

Our University Hospital of Northern Norway, situated at the 69.65° N latitude, has a large hospital catchment area though it mostly covers rural lands and is the only institution north of 63.43° N equipped with ECLS for rewarming. There are <500,000 inhabitants of which most are spread out in small communities along an intricate coastline of islands and fjords. Thus, the transport time for accidental hypothermia patients is sometimes substantial, which makes evacuation and transportation time typically 3–4 h for patients with HCA in need of in-hospital rewarming. When transport to our ECLS facility is impossible, mostly due to weather conditions, the local guidelines from 2014 suggest some form of thoracic lavage rewarming as an alternative option. This is recommended in a much-sited review of accidental hypothermia from 2012 (Brown et al., 2012), as well as in the American Heart Association guidelines (Nichol et al., 2010; Perkins et al., 2015). Closed thoracic (pleural) lavage may be instituted by the physicians in any local hospital (Brunette et al., 1987; Plaisier, 2005), utilizing the pleural cavity for infusion of warm saline, or tap water, to rewarm the heart and blood in central blood vessels by means of conduction and convection. However, the efficacy of closed thoracic lavage in rewarming accidental hypothermia patients is not explored experimentally.

In a porcine model of accidental hypothermia, we recently documented (Scientific Reports in press) that rewarming with ECLS following a 3-h period of continuous CPR for HCA at 27°C, took over global hemodynamic function, restored blood flow to the heart and the brain, and created a “shockable” cardiac rhythm. In the present study, we used the same experimental protocol to rewarm animals following a 3-h period of continuous CPR for HCA at 27°C by use of continuous closed thoracic lavage.

## Materials and Methods

The Norwegian Food Safety Authority approved the study (ID 5748, internal reference: 33/13). In this study, 11 castrated male pigs (wt. 26 ± 4 kg, age 3 months) from NOROC stock were used. On arrival, the animals were acclimated for 2–5 days before the terminal experiment. The animals were fed two times daily, always had free access to water, and received humane care in accordance with the Norwegian Animal Welfare Act.

### Anesthesia and Instrumentation

We previously reported the detailed methods for hemodynamic monitoring, immersion cooling, and blood flow measurements using the porcine animal model (Valkov et al., 2019). Briefly, after fasting the animals overnight, premedication was induced by an intramuscular bolus of ketamine hydrochloride (20 mg kg^−1^), midazolam (30 mg), and atropine (1 mg), and anesthesia was induced by a bolus infusion of fentanyl (10 μg kg^−1^) and pentobarbital-sodium (10 mg kg^−1^) in an ear vein. After tracheotomy and intubation, the animals were connected to a respirator (Siemens Servo 900D, Solna, Sweden), adjusted to maintain PaO_2_ > 10 kPa and PaCO_2_ at 4.5–6.0 kPa uncorrected for temperature (α-stat management). During the ventricular fibrillation and CPR, FiO_2_ was set to 1.0, infusion of fentanyl (20 μg kg^−1^ h^−1^) and midazolam (0.3 μg kg^−1^ h^−1^). Pentobarbital-sodium (4 mg kg^−1^ h^−1^) was continued *via* a femoral vein catheter. Anesthesia was discontinued during cooling at 27°C and re-instituted at the start of rewarming. The microspheres were injected into the left ventricle through a 6F fluid-filled pigtail catheter, (Cordis Corporation, Miami, FL, USA). The core temperature, CO, and venous and mixed venous blood gases were measured *via* a 7F pulmonary artery thermodilution catheter (Edwards Lifesciences LLC, Irvine, CA, USA) positioned in the pulmonary trunk. Thermodilution is described as the gold standard for measuring CO during CPR (Carretero et al., 2010). The tip of another 7F pulmonary artery thermodilution catheter was positioned in the aortic arch *via* the left femoral artery to monitor the arterial blood gas and mean arterial pressure (MAP) and to collect the reference blood samples for the microsphere technique. A 3.5F pressure catheter (SPR-524, Millar Instruments Inc., Huston, TX, USA) for monitoring of intracranial pressure (ICP) was placed in the brain parenchyma just below the dura mater through a 2 mm cranial hole drilled 1 cm to the right of the sagittal suture and 1 cm dorsal to the coronal suture. The urinary output was followed by a 14F urinary bladder catheter introduced *via* a lower abdominal incision. For rewarming, two PVC tubes were placed in the left pleural space, one (16F) in the mid-clavicular line in the second intercostal space, the other (24F) in the mid-axillary line in the sixth intercostal space. The animals were given 5,000 IE Heparin and allowed to stabilize for 45 min before the start of the experimental protocol.

### Regional Blood Flow Measurements

To determine organ blood flow, at each sampling point, ~10 million 15μm microspheres labeled with different stable isotopes (BioPAL Inc., Worcester, MA, USA) were injected into the left ventricle (Reinhardt et al., 2001). The reference blood samples were drawn from the aortic arch (5ml min^−1^, 2min) simultaneously with microsphere injections to calculate the regional blood flow. Blood flow was determined in the tissue samples from the brain (temporal lobes and cerebellum), kidneys, liver, heart, small intestine, spleen, and stomach based on a technique of neutron activation to analyze the microsphere content as already described in detail (Valkov et al., 2019).

### Experimental Protocol

**Figure 1:**
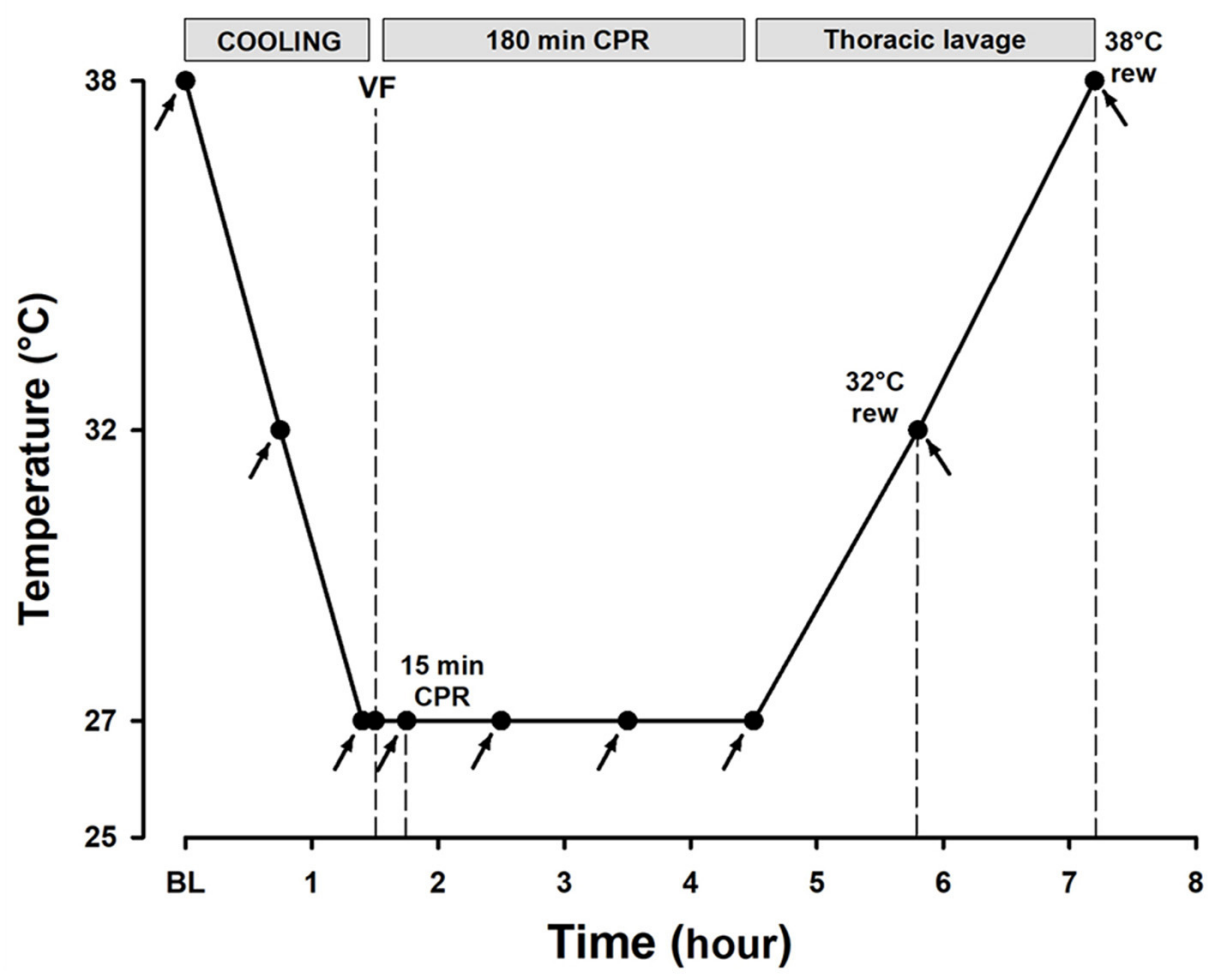
Experimental timeline. Arrows depict the time points for hemodynamic measurements, blood sampling, and microsphere administration.

The animals were immersion-cooled in ice water to a blood temperature of 27°C, and ventricular fibrillation was induced by stimulating the epicardial surface using an alternating current (5–20 mA, 6 Hz, and 30 V) delivered *via* a 15-cm-long needle electrode (Figure 1). The needle was inserted in the epigastric area, pointed toward the heart apex. Correct needle placement was confirmed when aspirating arterial blood from the left ventricle. Cardiac arrest was defined as asystole or ventricular fibrillation observed by ECG with an associated absence of fluctuation in arterial pressure. After 90 s of cardiac arrest, an automated chest compression device (LUCAS chest compression system, Physio-Control Inc., Lund, Sweden) was started. The piston on this compression device was equipped with a suction cup to ensure active decompression with a continuous mode compression/decompression duty cycle of 50 ± 5% at a rate of 100 ± 5 compressions/min and compression depth was 4–5 cm. For rewarming to 38°C after a 3-h period of CPR at 27°C, *via* tubing, the upper (inlet) pleural tube was connected to a roller pump circulating water at 40–42°C from the reservoir, whereas drainage was obtained *via* the outlet tube by gravity. Care was taken to keep the flow rate <500 ml min^−1^ to avoid a potential tension hydrothorax (Barr et al., 1988), with close attention to the water temperature and water level in the reservoir. Based on the previous observation of multiple costae and sternal fractures during a 3-h period of CPR using the LUCAS chest compression system (Nilsen et al., 2020), if cardioversion was unsuccessful after the three shocks at 30°C, a sternotomy was made to evacuate extravascular blood, followed by internal defibrillation (5–15 J). The experiment was concluded after core body temperature reached 38°C and the animals were euthanized.

### Rewarming With Closed Thoracic Lavage

During continuous CPR above 30°C, the appearance of spontaneous “shockable rhythms” in the ECG would initiate attempts with cardioversion, precipitated by intra-cardiac bolus injections of epinephrine (0.05–0.25 mg) to maintain MAP above 35 mmHg. If there was no cardioversion at 30°C after three repeated attempts at 50, 100, and 150 J, the procedure was repeated when core body temperature reached 31–32°C or 33–34°C. In the case of persistent arrhythmias, amiodarone (100–150 mg) was injected through intravenous (IV) followed by up to three additional cardioversion attempts (at 100 J).

A perfusing rhythm was defined following the cardioversion attempts at >30°C as the appearance of spontaneous pulsations in the arterial pressure wave with a MAP >35 mmHg lasting >10 min, accompanied by a sinus rhythm in the ECG. Epinephrine infusion was instituted to maintain MAP > 35 mmHg during rewarming.

### Measurements and Sampling Rates

The mean arterial pressure (MAP), left ventricular pressure, heart rate (HR), intracranial pressure (ICP), central venous pressure (CVP), were recorded using PowerLAB 16/35 and LabChart software (ADInstruments, Dunedine, New Zealand). CO was measured by thermodilution using 10 ml cold saline injected into the PA-catheter and recorded on a Vigilance monitor (Edwards Lifesciences, Irvine, CA, USA). All the measurements were obtained at three core body temperatures: baseline 38°C, during cooling at 32°, and at 27°C. The measurements were also obtained during CPR after 15, 60, 120, and 180 min, and during rewarming at 32° and 38°C.

### Statistics

The statistical analyses were performed using Sigma Plot statistical software version 13 [Systat Software Inc. (SSI), Richmond, CA, USA]. A Sample size was calculated using power analysis. Normal distribution was assessed using the Shapiro–Wilk test. Intragroup comparisons were performed by one-way repeated measures ANOVA for normally distributed variables, and Friedman's repeated measures ANOVA on ranks for non-normally distributed variables. Where significant differences were found, Dunnett's test was used to compare the values within group vs. baseline. The level of significance was set at *p* < 0.05. Data are presented as means and SD.

## Results

At the start of the experimental protocol, three animals died within the first 90 min of CPR due to physical damage to the atria by the tip of the lavage catheters causing hemothorax. After careful placement of the lavage catheters further out of reach for the piston on the automated chest compression device, this problem was eliminated, and as per protocol, only data from the other eight animals are included.

### Immersion Cooling and 3-h Period of CPR at 27°C

#### Hemodynamic Changes

**Figure 2:**
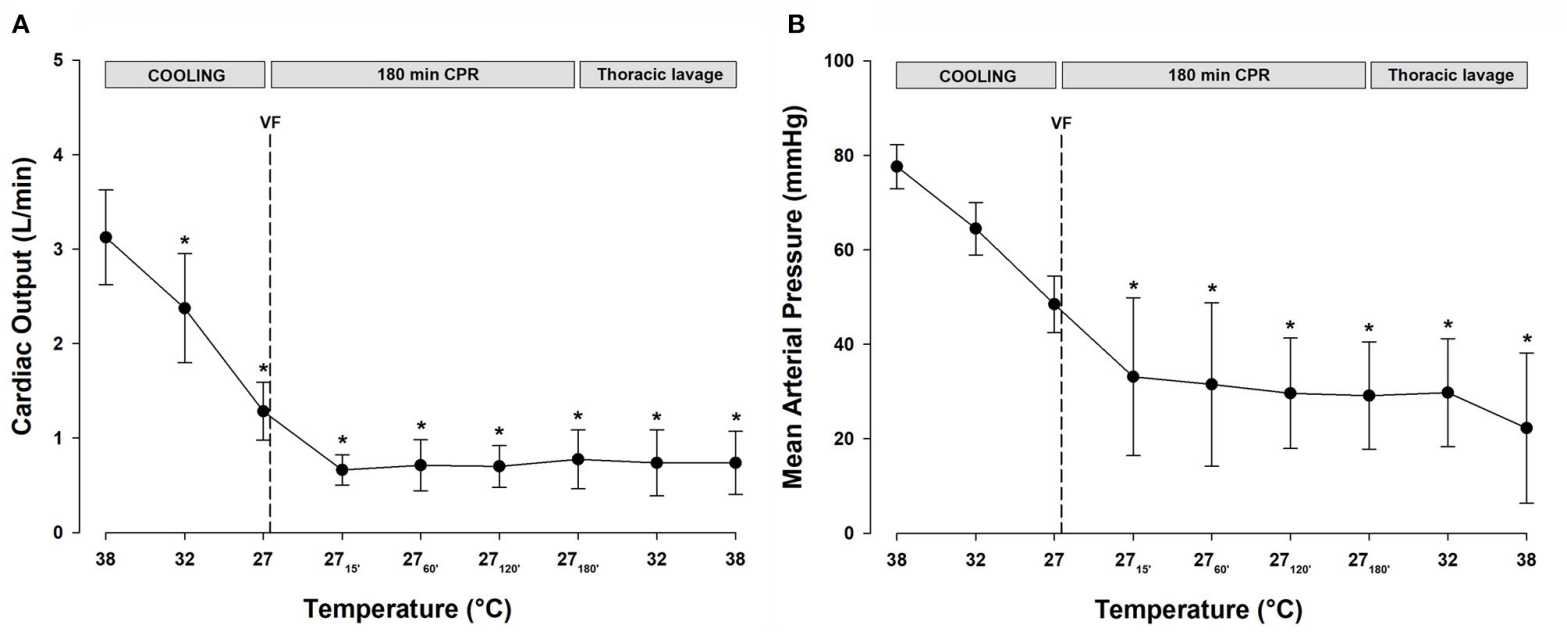
**(A)** Cardiac output. **(B)** Mean arterial pressure. *n* = 8, values are mean ± SD. *p < 0.05 statistically significantly different from the baseline value.

All statistical comparisons are reported in reference to the individual baseline (38°C) values (Figures 2A,B). Cooling reduced MAP (Figure 2B, Table 1) significantly from 78 ± 5 to 49 ± 6 mmHg at 27°C (−37%). After 15 min of CPR, MAP fell further to 33 ± 17 mmHg (−58%) and remained at this reduced level throughout the remaining 3-h period of CPR. Similarly, after cooling to 27°C, CO (Figure 2A, Table 1) fell significantly from 3.1 ± 0.5 to 1.3 ± 0.3 L min^−1^ (−58%). After 15 min of CPR, CO fell even further to 0.7 ± 0.2 L min^−1^ (−77%) and remained at this reduced level during the remaining 3-h period of CPR.

**Table 1:** Hemodynamic variables and values for cerebral pressures.

	**38^**°**^C**	**27^**°**^C**	**27^**°**^C_**15 min**_**	**27^**°**^C_**3-h**_**	**RW 32^**°**^C**	**RW 38^**°**^C**
MAP, mmHg	78 ± 5	49 ± 6^*^	33 ± 17^*^	29 ± 11^*^	30 ± 11^*^	22 ± 16^*^
PAP, mmHg	18 ± 3	20 ± 4	42 ± 22^*^	41 ± 15^*^	30 ± 12	29 ± 9
LVSP, mmHg	114 ± 10	68 ± 10^*^	108 ± 59	119 ± 46	84 ± 44	73 ± 33
CVP, mmHg	5 ± 2	5 ± 2	37 ± 27^*^	30 ± 18^*^	23 ± 14^*^	23 ± 15^*^
ICP, mmHg	13 ± 3	13 ± 5	16 ± 3	16 ± 5	17 ± 4^*^	19 ± 2^*^
CPP, mmHg	64 ± 6	26 ± 18^*^	17 ± 15^*^	15 ± 10^*^	14 ± 10^*^	6 ± 14^*^
HR, 1/min	98 ± 14	62 ± 16^*^	100 ± 0	100 ± 0	100 ± 0	100 ± 0
CO, L/min	3, 1±*0, 5*	*1, 3*±0, 3^*^	*0, 7*±0, 2^*^	*0, 8*±0, 3^*^	*0, 7*±0, 4^*^	*0, 7*±0, 3^*^
SV, ml	32 ± 5	21 ± 3^*^	7 ± 2^*^	8 ± 3^*^	7 ± 4^*^	7 ± 3^*^
TPR, mmHg/L/min	25 ± 3	39 ± 9	54 ± 35^*^	43 ± 23	45 ± 22	32 ± 15

*MAP, mean arterial pressure; PAP, pulmonary arterial pressure; LVSP, left ventricular systolic pressure; CVP, central venous pressure; ICP, intracranial pressure; CPP, cerebral perfusion pressure (CPP = MAP – ICP); HR, heart rate; CO, cardiac output; SV, stroke volume (SV = CO/HR); TPR, total peripheral resistance (TPR = MAP/CO). n = 8, values are mean and SD.*

* *p < 0.05 statistically significantly different from the baseline value*.

#### O_2_ Transport and Extraction

**Figure 3:**
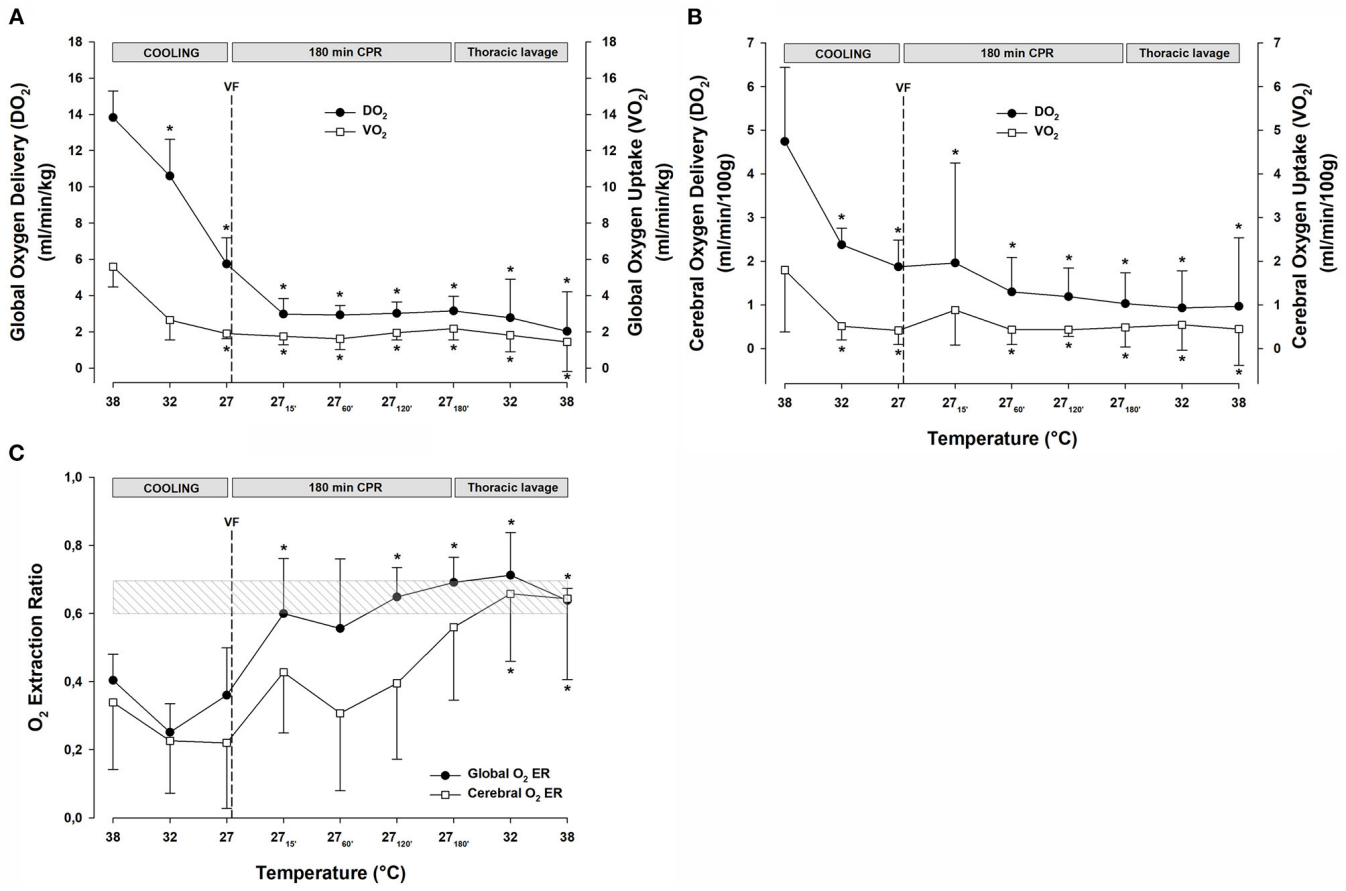
**(A)** Global oxygen delivery, and global oxygen uptake (VO_2_). **(B)** Cerebral oxygen delivery, and cerebral oxygen uptake (VO_2_). **(C)** Global and cerebral oxygen extraction ratio. *n* = 8, values are mean ± SD. *p < 0.05 statistically significantly different from the baseline value. Striated area indicates a critical level of extraction ratio.

Global DO_2_ (Figure 3A) was reduced significantly during cooling to 27°C from 13.8 ± 1.5 to 5.7 ± 1.4 ml min^−1^ kg^−1^ (−59%). Similarly, VO_2_ (Figure 3A) decreased during cooling to 27°C from 5.6 ± 1.1 to 1.9 ± 0.3 ml min^−1^ kg^−1^ (−66%). After 15 min of CPR, DO_2_ was further reduced to 3.0 ± 0.9 (−78%), and VO_2_ was reduced to 1.7 ± 0.5 (−70%) ml min^−1^ kg^−1^, and both DO_2_ and VO_2_ remained at these reduced levels throughout the remaining 3-h period of CPR. Global O_2_ extraction ratio (DO_2_/VO_2_) (Figure 3C) was significantly reduced by cooling to 27°C, and after 60 min of CPR, the extraction ratio reached 0.69 ± 0.07, the reported critical extraction ratio (ER_crit_) necessary to provide aerobic metabolism (Fairley et al., 1957). Due to the stable CO throughout the 3-h period of CPR period, the extraction ratio remained at this elevated level. Cerebral DO_2_ (Figure 3B) was reduced significantly during cooling to 27°C from 4.7 ± 1.7 to 1.9 ± 0.6 ml min^−1^ (100 g)^−1^ (−60%). Cerebral VO_2_ (Figure 3B) decreased significantly during cooling to 27°C from 1.8 ± 1.4 to 0.4 ± 0.3 ml min^−1^ (100 g)^−1^ (−78%). After 15 min of CPR, cerebral DO_2_ remained at the same level as at cooling 27°C, while cerebral VO_2_ increased to 0.9 ± 0.8 ml/min/100 g. Both the cerebral DO_2_ and VO_2_ remained significantly reduced throughout the 3-h period of CPR.

#### Arterial Lactate, pH, and Central Venous O_2_ Saturation (SvO_2_)

Cooling to 27°C and the 3-h period of CPR caused an almost linear reduction in pH (Table 2) from 7.53 ± 0.05 to 7.26 ± 0.08, simultaneously with an increase in serum lactate (Table 2) from 0.8 ± 0.2 to 5.0 ± 1.8 mmol L^−1^. These relatively modest changes in pH and lactate took place while SvO_2_ (Table 2) fell from 59 ± 8 to 31 ± 10%.

**Table 2:** Blood gases and biochemical variables.

	**38^**°**^C**	**27^**°**^C**	**27^**°**^C_**15 min**_**	**27^**°**^C_**3-h**_**	**RW 32^**°**^C**	**RW 38^**°**^C**
pH	7.53 ± 0.05	7.45 ± 0.02*	7.4 ± 0.04*	7.26 ± 0.08*	7.15 ± 0.08*	6.97 ± 0.17*
Hb (g/dL)	8.4 ± 0.3	8.8 ± 0.5	8.5 ± 0.6	8.6 ± 0.7	8.8 ± 0.7	7.6 ± 1.7
Hct (%)	26 ± 1	27 ± 2	26 ± 2	27 ± 2	28 ± 3	24 ± 5
Lactate (mmol/l)	0.8 ± 0.2	0.8 ± 0.7	1.7 ± 1.0*	5.0 ± 1.8*	8.1 ± 2.2*	10.0 ± 3.9*
BE (mmol/l)	4.7 ± 2.0	4.1 ± 1.6	−0.6 ± 2.9*	−6.6 ± 2.2*	−12.9 ± 4.1*	−18.6 ± 5.9*
HCO_3_- (mmol/l)	29 ± 2	28 ± 1	24 ± 3*	19 ± 2*	14 ± 3*	10 ± 5*
K^+^ (mmol/l)	3.3 ± 0.4	2.7 ± 0.5	3.1 ± 0.8	4.9 ± 1.3*	6.0 ± 1.7*	7.4 ± 2.8*
PaO_2_ (kPa)	15 ± 7	18 ± 2	25 ± 23	19 ± 18	16 ± 20	10 ± 9
PaCO_2_ (kPa)	4.5 ± 0.5	5.4 ± 0.5	5.0 ± 0.9	6.0 ± 1.4	5.7 ± 1.5	6.5 ± 2.2
SaO_2_ (%)	99 ± 2	99 ± 1	95 ± 6	91 ± 8	76 ± 21*	57 ± 26*
SvO_2_ (%)	59 ± 8	67 ± 15	41 ± 18*	31 ± 10*	24 ± 15*	17 ± 9*
SvO_2_ jug.bulb (%)	65 ± 15	87 ± 12*	57 ± 16	41 ± 20*	21 ± 15*	18 ± 15*

Hct, hematocrit; BE, base excess; HCO_3_-, bicarbonate; P_a_O_2_, arterial partial O_2_ pressure; P_a_CO_2_, arterial partial CO_2_ pressure; SaO_2_, arterial O_2_ saturation; SvO_2_, mixed venous O_2_ saturation; SvO_2_ jug.bulb., jugular bulb venous O_2_ saturation. n = 8, values are mean and SD. *p < 0.05 statistically significantly different from the baseline value.

#### Organ Blood Flow

**Figure 4:**
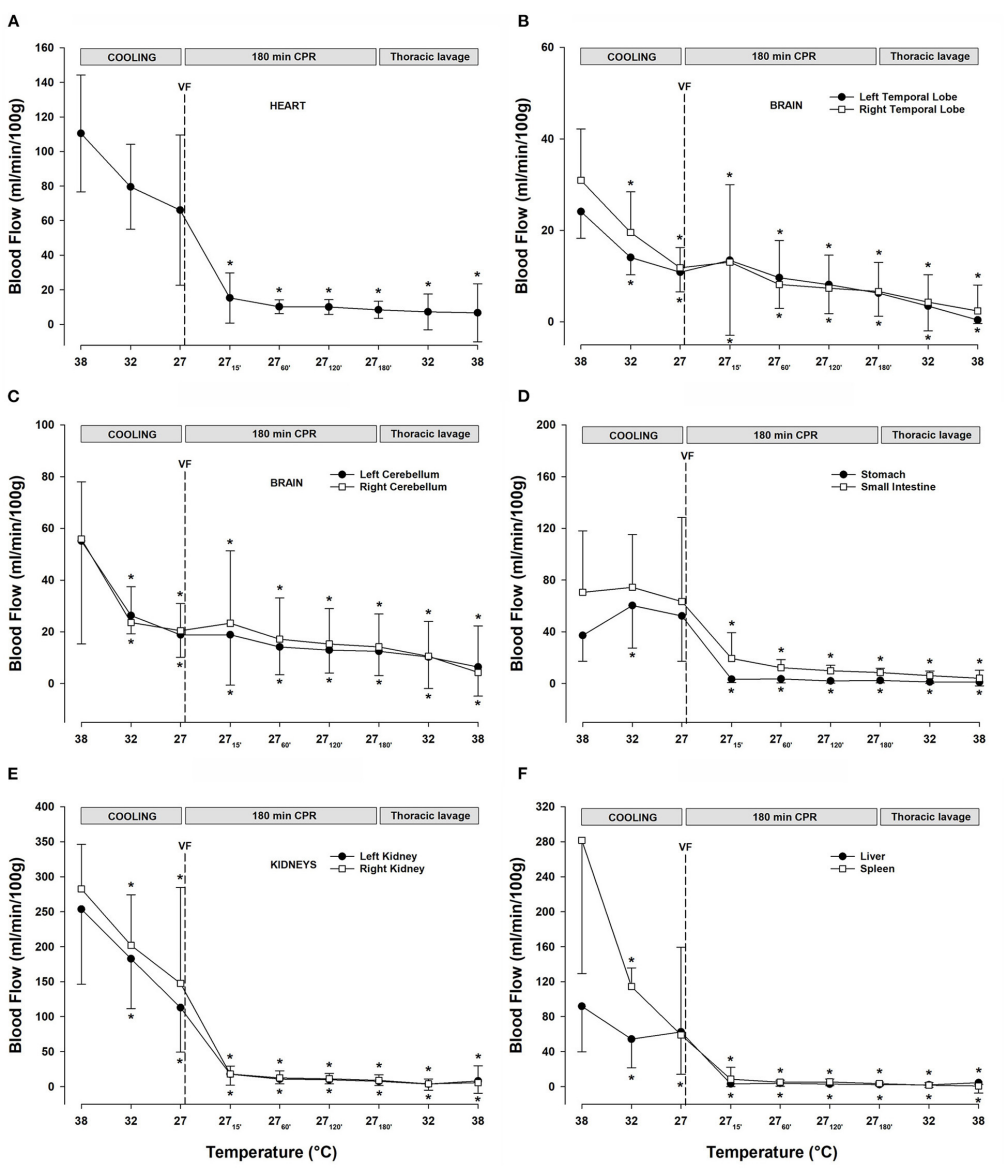
**(A)** Myocardial blood flow. **(B)** Blood flow in the left and right temporal lobes. **(C)** Blood flow in the left and right cerebellar hemispheres. **(D)** Blood flow in the stomach and small intestine. **(E)** Renal blood flow. **(F)** Blood flow in the liver and the spleen. *n* = 8, values are mean ± SD. *p < 0.05 statistically significantly different from baseline value.

After ventricular fibrillation and 15 min CPR at 27°C, myocardial blood flow (Figure 4A) was significantly reduced compared with the baseline (−86%) and remained at this reduced level during the remaining 3-h period of CPR. After cooling to 27°C, blood flow in the temporal lobes (Figure 4B) was significantly reduced (left lobe −54%, and right lobe −61%). Compared with the baseline, after 15 min of CPR, blood flow to the left and right temporal lobe remained reduced, by −46 and −38%, respectively, but after the 3-h of CPR, blood flow to the left and the right temporal lobes were further reduced to −75 and −77% of baseline, respectively. Cooling to 27°C significantly reduced the blood flow to the left (−65%) and right (−64%) cerebellar hemispheres (Figure 4C). Initially, after 15 min of CPR, there was no further reduction in the cerebellar blood flow, but after 3-h of CPR, blood flow to the left (−78%) and the right (−75%) cerebellar hemispheres was further reduced. The abdominal organs (Figures 4D-F) showed a varying reduction in the blood flow during cooling, as well as during a 3-h period of CPR. Cooling to 27°C significantly reduced the blood flow to the left (−55%) and right (−48%) kidneys (Figure 4E), and renal blood flow was almost completely shut off after 3-h of CPR (-97%, both). Liver blood flow (Figure 4F) was unaltered after cooling to 27°C, whereas blood flow to the spleen (Figure 4F) was reduced (−79%). Both the organs had severely impaired blood flow during CPR, and after 3-h, the blood flow was reduced by −98% in the liver and −99% in the spleen compared with baseline at 38°C. After cooling to 27°C, the blood flow to the stomach and small intestine (Figure 4D) remained statistically unchanged. However, after 3-h of CPR, blood flow to the stomach and small intestine was severely reduced by −95 and −88% of baseline, respectively.

### Rewarming With Closed Thoracic Lavage

Lavage rewarming gave a mean rewarming rate of 3.8°C h^−1^ (max 12°C h^−1^ and min 1.6°C h^−1^). The total volume of IV Ringer infused was 1.7 ± 0.4 l.

#### Attempts to Achieve a Perfusing Rhythm

Based on continuous ECG monitoring, all the animals had ventricular fibrillations during CPR and CPR/rewarming. At 30°C, attempts to achieve a perfusing rhythm were made by cardioversion of ventricular fibrillation to sinus rhythm. In only one out of eight animals, a perfusing rhythm (sinus rhythm, MAP between 49 and 60 mmHg) was achieved at 31°C after the two cardioversion attempts (50 and 100 J), and a single bolus of 0.1 mg epinephrine. The three animals had a spontaneous conversion from ventricular fibrillation to ventricular tachycardia during rewarming above 30°C and were treated with repeated IV injections of amiodarone (100–150 mg) followed by unsuccessful cardioversion attempts (100 J, up to three times). In the remaining four animals, the repeated attempts to convert the ventricular fibrillation were unsuccessful at all predetermined temperatures.

#### Hemodynamic Function

Throughout the period of lavage rewarming and continuous CPR, CO, and SV (Table 1) remained lowered at 27°C, and after rewarming they remained reduced at −80 and −79%, respectively, when compared with the baseline values at 38°C. In the single animal where a perfusing rhythm was established under continuous epinephrine infusion, MAP remained at 50–60 mmHg, whereas CO was reduced, −77%, when compared with its baseline value.

#### O_2_ Transport and Extraction

The continuous CPR and closed thoracic lavage rewarming did not change global DO_2_ (Figure 3A), and at 38°C, it was significantly reduced compared with the baseline [15.2 ± 4.4 vs. 9.4 ± 3.0 ml min^−1^ (100 g)^−1^; −38%]. Likewise, global VO_2_ (Figure 3A) remained unchanged during the closed thoracic lavage rewarming, and at 38°C, it was significantly reduced compared with the baseline [6.8 ± 2.5 vs. 4.3 ± 1.1 ml min^−1^ (100 g)^−1^; −34%].

During rewarming, the cerebral DO_2_ and VO_2_ (Figure 3B) remained unaltered but the difference between them appeared practically vanished at 38°C, and they were both significantly reduced when compared with the baseline (DO_2_: 4.41 ± 2.14 vs. 2.11 ± 2.23; −50% and VO_2_: 2.08 ± 1.17 vs. 0.85 ± 0.87 ml min^−1^ (100 g)^−1^; −56%). Both the global and cerebral extraction ratios (Figure 3C) increased to values ~0.7 during rewarming and at 38°C, they were significantly elevated compared with the baseline values.

During rewarming at 32 and 38°C, a substantial reduction in arterial pH (Table 2) from 7.15 ± 0.08 to 6.97 ± 0.17, respectively, was measured, and both the values were significantly reduced compared with the 38°C baseline pH value of 7.53 ± 0.05. The reduced pH occurred simultaneously with a significant increase in serum lactate (Table 2) from the baseline 38°C value of 0.8 ± 0.2 to 10.0 ± 3.9 mmol L^−1^ after rewarming, a significant reduction of ${\text{HCO}}_{3}^{-}$ (Table 2) from 29 ±2 to 10 ± 5 mmol L^−1^, and a fall in S_v_O_2_ (Table 2) from 59 ± 8 to 17 ± 9%.

#### Organ Blood Flow

Lavage rewarming did not lead to any change in the already reduced blood flow (Figures 4A-F) measured during CPR at 27°C in all organs investigated.

## Discussion

In the present study, a porcine experimental animal model was used to mimic the condition of prehospital accidental hypothermia with HCA and CPR for a 3-h period at 27°C. Specifically, we explored if rewarming by closed thoracic lavage could be used to establish a spontaneous “shockable” cardiac rhythm and reestablish a perfusing rhythm. The potential translational value of this experiment was to provide evidence supporting (or not) the use of closed thoracic lavage rewarming after long-lasting CPR in the accidental hypothermia patients in a local hospital as an alternative to continued CPR and transportation to a medical center equipped for rewarming with ECLS. The safe time limits for hypothermic CPR are yet not documented, whereas it is well-documented that during normothermic CPR, it is vital to establish a perfusing rhythm within 20 min. The present study demonstrated that the continuous CPR at 27°C maintained CO, MAP, and blood flow to the vital organs at the same reduced level for 3-h and supported global aerobic metabolism. However, the ensuing rewarming using closed thoracic lavage and continued CPR failed to establish an adequate perfusing rhythm in any animal.

All the animals in this study had ventricular fibrillation during rewarming and attempts to establish a perfusing rhythm were started at 30°C by cardioversion. We were able to establish a sinus rhythm in only one out of eight animals during rewarming, but due to electro-mechanic dissociation, it was out of reach to establish a perfusing rhythm. In all animals, the attempts to establish a perfusing rhythm were under continuous epinephrine support, which re-established MAP (50–60 mmHg) but failed to elevate CO that remained seriously reduced (−77%). After rewarming by closed thoracic lavage, the reduced CO was unable to elevate the organ blood flow or DO_2_ over the corresponding values measured during CPR at 27°C. During the period of rewarming by closed thoracic lavage, all the animals had seriously reduced organ blood flow and simultaneously worsening metabolic acidosis. The failure to re-establish perfusion ended in circulatory collapse at 38°C. In a previous report, we documented that the level of CO produced by CPR during 38°C remained unchanged after cooling to 27°C (Nilsen et al., 2020). The results from the present experiment support this observation, and consequently, the low CO produced by continuous CPR must be converted to a perfusing rhythm during rewarming at 30–32°C to make closed thoracic lavage an alternative to ECLS as a rewarming technique. Further, in the single animal where ventricular fibrillation was converted to a perfusing rhythm, the CO remained low despite pharmacologic support. This observation is consistent with the existence of hypothermia-induced, left ventricular dysfunction (Bigelow et al., 1950; Fairley et al., 1957; Tveita et al., 1994, 1998; Filseth et al., 2010), which may necessitate massive cardiac support, making ECLS the only therapeutic alternative. So far, the longest period of HCA before successful resuscitation and rewarming with ECLS has been reported after 6.5 h of continuous CPR (Lexow, 1991). In the porcine model, we recently documented (Scientific Reports in press) successful rewarming by ECLS after a 3-h period of continuous CPR using an extracorporeal membrane oxygenator (ECMO), which restored the blood flow to the heart and the brain. After the period of CPR and rewarming by ECLS, we were able to convert the spontaneous ventricular fibrillation to a sinus rhythm in all the animals. Successful rewarming by ECLS after long-lasting CPR for HCA has been documented (Brown et al., 2012), but after rewarming, these patients often need cardio-respiratory support for days, a fact in favor of using ECMO-rewarming (Ruttmann et al., 2007). In fact, ECMO rewarming has been suggested to be superior to other ECLS techniques (Silfvast and Pettila, 2003). By using the ECMO technique for rewarming, the survival and neurologic outcome of accidental hypothermia patients with HCA has improved significantly (Debaty et al., 2017; Bjertnaes et al., 2021).

### Thoracic Lavage

Technically, the thoracic lavage is applicable as open or closed. Open thoracic lavage involves a thoracic incision big enough to enable manipulation of the heart and other mediastinal structures, while directly pouring warm liquid on the heart (Linton and Ledingham, 1966; Althaus et al., 1982; Best et al., 1985; Brunette et al., 1992). Closed thoracic lavage, as used in this experiment, involves small incisions to enable the insertion of one or more pleural drains in one or both thoracic cavities. This procedure is often performed by inserting one “inlet” drain and one “outlet” drain in the left thoracic cavity (Hall and Syverud, 1990; Iversen et al., 1990; Kornberger et al., 2001; Schwarz et al., 2002), and the procedure can be performed by a variety of hospital physicians (Brunette et al., 1987; Plaisier, 2005). With closed thoracic lavage, external chest compressions can be performed throughout the resuscitation. This may be beneficial as it preserves the thoracic pump mechanism (Plaisier, 2005), one of the key factors for a favorable outcome during CPR, linked to the recognition of chest wall recoil and negative thoracic pressure as mechanisms for venous blood return, as well as for cerebral- and coronary blood flow in the decompression phase (Georgiou et al., 2014). However, a feared consequence of the closed thoracic lavage is an increase in intrathoracic pressure. This unwanted side effect is most often brought about by a mismatch between inlet and outlet volumes leading to the retention of water and subsequent increase in pressure inside the thoracic cavity. Any increase in thoracic pressure may cause dramatic consequences on the hemodynamic function during spontaneous circulation, as well as during CPR (Aufderheide et al., 2008; Metzger et al., 2012; Kill et al., 2014).

There are only a few human reports (Winegard, 1997; Plaisier, 2005; Kjaergaard and Bach, 2006; Turtiainen et al., 2014) and animal studies (Brunette et al., 1987; Barr et al., 1988; Otto and Metzler, 1988), utilizing closed thoracic lavage in the context of rewarming from hypothermia and HCA, with no conclusions regarding the superiority of one rewarming technique over the other. Further, the longest period of HCA before successful resuscitation and rewarming with closed thoracic lavage so far is 3 h and 13 min (Winegard, 1997). Thoracic lavage is independent of an intact circulation to effectively warm the heart and is, except for ECLS, probably among the fastest active internal rewarming techniques (Brown et al., 2012) to facilitate defibrillation and to establish a perfusing rhythm in a patient with HCA (Plaisier, 2005). In addition, in hypothermic patients with unstable circulation, direct rewarming of the heart with this method may be protective against ventricular fibrillation (Barr et al., 1988).

Despite many unanswered questions related to using closed thoracic lavage, the introduction of its use in humans took only a couple of years as described in a case report (Hall and Syverud, 1990). ECLS is not always available for rewarming patients in HCA; thus, closed thoracic lavage was suggested as an alternative method that appears to be both efficient and safe when applied by competent physicians (Winegard, 1997; Plaisier, 2005; Kjaergaard and Bach, 2006; Turtiainen et al., 2014). However, to recommend this method as an alternative to ECLS for a variety of physicians to rewarm accidental hypothermia patients in any local hospital (Brunette et al., 1987; Plaisier, 2005), it is essential that evidence of its utility must be demonstrated as documenting any potential negative side effects. Unfortunately, the evidence base for the application of closed thoracic lavage as an alternative to ECLS was never provided.

In this study model, it often appeared challenging to combine the use of closed thoracic lavage with ongoing CPR. The practical challenges were many, such as maintaining constant inflow temperature and constant tap water flow, maintaining a closed circuit, and preventing a build-up in intrathoracic pressure. We previously have documented challenges related to the use of closed thoracic lavage for rewarming during spontaneous circulation (Valkov et al., 2019), without simultaneous CPR. In sum, the spread in rewarming rate using closed thoracic lavage documented in the present experiment may be taken as the consequence of all these practical issues.

### Continuous CPR

In addition to the factors described above, it is possible that prolonged CPR causes trauma to the heart. However, in the 82 retrospective cases of CPR for 20–230 min, only 10 patients were treated for HCA and only seven cases had reduced ejection fraction. Of these seven cases, one patient had aortic valve deformity, and there were no other cardiac CPR-related complications reported (Youness et al., 2016).

## Conclusion

Anecdotal case reports have suggested that the accidental hypothermia patients can be successfully rewarmed with thoracic lavage, but with little if any documentation. Our animal experiment, evaluating the effects of rewarming by closed thoracic lavage after a 3-h period of continuous CPR for HCA at 27°C, found that the critical element of the successful use of this method, reestablishment of a perfusing rhythm during the early part (30–32°C) of rewarming, could not be demonstrated. Thus, the results of this experiment do not support the use of closed thoracic lavage as an alternative to ECLS for rewarming accidental hypothermia patients after long-lasting CPR for HCA.

## Data Availability Statement

The raw data supporting the conclusions of this article will be made available by the authors, without undue reservation.

## Ethics Statement

The animal study was reviewed and approved by The Norwegian Food Safety Authority.

## Author Contributions

TT, GS, JN, RM, and TK contributed to the conception and design, data analysis and interpretation, and drafting the manuscript for intellectual content. JN, RM, TS, JN, SV, and TK contributed to the completion of experiments and collection of data. JN, RM, TS, JN, SV, TK, GS, and TT contributed to the revision of the manuscript. All authors contributed to the article and approved the submitted version.

## Funding

This work was generously supported by grants from the Norwegian Research Council (Petromax2) and the Norwegian Ministry of Foreign Affairs (Barents 2020).

## Conflict of Interest

The authors declare that the research was conducted in the absence of any commercial or financial relationships that could be construed as a potential conflict of interest.

## Publisher's Note

All claims expressed in this article are solely those of the authors and do not necessarily represent those of their affiliated organizations, or those of the publisher, the editors and the reviewers. Any product that may be evaluated in this article, or claim that may be made by its manufacturer, is not guaranteed or endorsed by the publisher.
